# Gynecology and Abdominal Surgery

**Published:** 1899-01

**Authors:** 


### GYNECOLOGY AND ABDOMINAL SURGERY.

**Papers Presented at the Memphis Meeting of the Southern Surgical aud Gynecological Association, Dec. 6-8, 1898.**

Dr. William E. Parker, of New Orleans, La., presented a paper entitled

### GUNSHOT WOUNDS.

He described the hospital conditions in Cuba and the wounds seen by him made by the modern bullet. He spoke of some experiments made under the direction of Major L. A. LaGarde, surgeon U. S. A., as to the relative penetrating powers of the Mauser, Krag-Jorgensen and Springfield rifles. With ammunition of the years 1896 and 1897 the Mauser, fired at a distance of three feet from the muzzle into seasoned yellow pine posts, would penetrate 34 + inches; with ammunition of 1895 it would penetrate 32 + inches; with ammunition of the past year, the Krag-Jorgensen, fired at the same distance, would penetrate 24 + inches, and the Springfield would penetrate but 5 inches; yet many lodged Mausers were found, and he was told by the Spanish chief surgeon that but six lodged Krag-Jorgensens were found by them. In speaking of wounds of special regions, he said that flesh wounds, as a rule, healed under one dressing. In the few cases that did not, the sloughing was at the points of entrance and exit, and not along the whole track of the bullet. Explosive effects were not seen by any of the surgeons. He stated that he had hoped to present some statistics as to the wounds of the war, but they could not be had. Wounds of the brain did well. He had heard of at least five cases that were still alive and doing well. But one case of wound of the spinal cord was operated upon and he was doing well. This patient had paralysis of both legs and of the bladder and rectum. Some spiculæ of bone had been removed from the cord At this time the patient has perfect use of the right leg and partial use of the left, and has perfect control of his bladder and rectum. He said that while he had not in any way modified his opinion that laparotomy should be done for abdominal wound in civil life, with the small modern bullet and lack of proper facilities this operation should not be attempted in the field, unless there should be symptoms of hemorrhage. About 50 per cent. of these cases recovered without operation. Chest wounds did well and caused but little trouble. In cases of wounded vessels, hemorrhage did not seem greater than in cases wounded by other bullets. When nerves were injured, nerve suture was not attempted on account of the rush of work, but he thought it should be attempted as soon as possible. None of the hospitals had sufficiently large number of surgeons or hospital corps men, but he spoke of the efficiency of those who were there.

Dr. F. W. McRae, of Atlanta, Ga., followed with a paper on

PENETRATING WOUNDS OF THE ABDOMEN, WITH REPORTS OF CASES.

At the outset, the essayist referred to a resolution passed unanimously by the Association at the Nashville meeting in 1896, to the effect that in all cases of penetrating wounds of the abdomen it is the duty of the surgeon to make an exploratory incision so as to definitely determine whether the viscera have been injured or not. Since then a few able surgeons have dissented from this opinion. The propriety of surgical interference in cases of penetrating gunshot wounds of the abdomen will depend on one of three things: 1, the general condition of the patient; 2, dangerous internal hemorrhage; 3, wounds of the stomach or intestines large enough to permit extravasation. He quoted from contributions to this subject by Senn, Nunez, and others, and said, from the conflict of opinion and advice given by these and various other surgeons, one is at a loss to know just how to proceed in many cases. His own convictions, based on sixteen cases, which he narrated, are positive. While he has the greatest admiration and respect for the surgical opinions of Senn, he should be recreant to his convictions were he to accept the proposition of non-interference laid kown by Senn in his recent contributions to the subject. In most of the cases which have come under his observation where grave lesions of the abdominal viscera have existed, the symptoms of internal injury were practically nil, and the positive signs of grave damage almost entirely absent. In conclusion, he reiterated his firm conviction that every case of penetrating wound of the abdomen, where there is reasonable doubt as to injury of the abdominal viscera, should be subjected to at least an exploratory operation. This enables the surgeon to either absolutely be sure that no injury has been done to the viscera, or to repair, as far as possible, such injuries when they exist. The mortality following a simple exploratory operation *per se* is so small as not to weigh against the much greater mortality that necessarily follows the expectant plan of treatment. In the cases he had seen, the general and local signs have been so misleading and enigmatic that prior to the operation a conclusion on his part as to internal conditions would have been entirely guesswork.

Dr. Horace H. Grant, of Louisville, Ky., contributed a paper on

PRACTICAL MANAGEMENT OF BULLET WOUNDS OF THE ABDOMINAL VISCERA.

Observation and experience with operators coming frequently in contact with this lesion have determined that penetrating wounds of the abdomen demand laparotomy at the earliest possible moment after diagnosis and thorough inspection of the entire region endangered. It is established that not only is the mortality in untreated wounds of the abdomen almost 100 per cent. where the intestines are perforated, and very high in wounds of the solid viscera, but it becomes less promising every advancing hour, until by the second day peritonitis sets in and destroys the hopefulness of the prospect. Within the past two years the speaker has operated upon four cases. In each case the patient was seen early, in no instance later than four hours. In three, resection of the intestine was necessary. Three of these patients recovered; the fourth died from septic peritonitis due to the escape of a large quantity of fecal matter before operation. Autopsy disclosed a perfect intestine.

These three papers were discussed together.

Dr. W. L. Robinson, of Danville, Va., said he had seen a good many cases of gunshot wounds of the abdomen, and nearly all of them died, either with or without operation. Early operative interference might save some of them.

Dr. G. S. Byown, of Birmingham, said he knew of several cases of gunshot wounds of the abdomen that occurred in and around Birmingham, that recovered without operation. Personally, he had operated on five cases, one of which recovered. In this case there was no perforation of the intestine. Undoubtedly, diarrhea and starvation, mentioned by Dr. Parker in his paper, have something to do with the favorable results. The intestines are most liable to be perforated when full.

Dr. Willis F. Westmoreland, of Atlanta, said the results of gunshot wounds of the abdomen on the battle-field should not be compared with those met with in civil life. It is not a question of operating, so far as fatal results are concerned, but the manner in which the operation is done.

Dr. W. E. B. Davis, of Birmingham, thought that Dr. Parker's paper was not an argument against operative procedures for abdominal wounds. Surgeons on the battle-field do not have the same facilities for operating on penetrating wounds of the abdomen that they do in civil practice, and which are necessary for the proper care of cases. If a man sustains an injury to the intestine, if his abdomen is not opened, death may be expected. In twenty or more cases operated on by him and his brother, all of the cases died that were seen twenty-four hours after the injury. This is not an argument against operative interfence, but against late operation. Statistics impress surgeons with the importance of early operation.

Dr. George A. Baxter, of Chattanooga, believes it is not necessary in many instances to open the abdomen in cases of gunshot wounds, and cited a number of cases that recovered without operative interference.

Dr. John T. Wilson, of Sherman, Texas, advocated early operation in cases where the surgeon is convinced that perforation has taken place. The character, technique and rapidity of the operation are very important. In operating, the intestines should be exposed as little as possible.

Dr. W. L Rodman, of Louisville, said that while he has pursued the practice of operating on penetrating gunshot wounds of the abdomen for the last ten years, after hearing the paper of Dr. Parker and reading the recent contributions of Senn and others, he wondered whether he as well as other surgeons may not have gone too far in operating in so many of these cases; at the same time, he does not wish to be understood as advising a masterly inactivity where the environments for operative interference are favorable.

Dr. Westmoreland said that, given a case of perforation of the intestine, particularly where there is extravasation of contents, instead of being masterly inactivity, it is criminal inactivity for a surgeon not to operate

Dr. Parker, in closing the discussion on his part, said that he had tried to make a clear distinction between the plan of treatment of gunshot wounds of the abdomen met with on the battle-field and those encountered in civil life. He is strongly opposed to laparotomy on the battle-field on account of the conditions that exist. In civil life, however, the thing to do in case of gunshot wounds of the abdomen, is to get in and out of the belly as soon as possible consistent with good work.

Dr. McRae believes that a large proportion of operations undertaken for gunshot wounds of the abdomen late will terminate fatally.

Dr. Grant stated that practically all cases operated on after twenty-four hours perish. It is impossible to diagnosticate perforation of the intestines with any degree of certainty. If a patient is seen within eight or ten hours after the receipt of a gunshot wound of the abdomen, under favorable surroundings, whether the bullet has penetrated the intestine or not, the skillful surgeon will not hesitate to make at least an exploratory operation, because it would not hurt the patient, but would probably save his life.

Dr. Howard A. Kelley, of Baltimore, Md., presented a paper entitled

### THE TREATMENT OF COMPLETE RUPTURE OF THE PERINEUM BY DISSECTING OUT THE SPHINCTER MUSCLE AND ITS DIRECT UNION BY BURIED SUTURES.

He said that the results of the best methods of the treatment of complete tears of the perineum are not entirely satisfactory in a large percentage of cases. The control over liquid motions and flatus is as a rule not secured immediately, and it is usually necessary to encourage the patient by telling her that she "will have to learn to control the muscle in the course of time." Such a control, more or less perfect, is gained in the course of several months. This defect in our present procedures is due to a faulty approximation of the sphincter ends which lie buried in a pit, and are therefore difficult to bring into accurate, firm apposition by sutures embracing a considerable quantity of tissue surrounding the sphincter ends. Dr. Kelley proposes, therefore, the deliberate dissection and freeing of the sphincter ends, drawing them out about one and a half centimeters from the tissues, cutting off the scarred ends, and effecting a direct union of the freshened ends by two or three catgut sutures. He was led to do this operation by his experience in a case which had been operated upon six times with a result which, judged by superficial appearances, was perfect, and yet the patient had no control over her bowel functions. He made a semi-lunar incision around the anterior periphery of the anus and found the right sphincter end buried in scar tissue in the median line, while that of the left side was ectopic and attached out under the ischial tuberosity. The sphincter ends were united directly by buried catgut sutures, and the skin wound closed and union took place *per primam*. In addition to these buried catgut sutures, a slinting suture of silkworm gut is passed through the middle of the sphincter near the edges of the wound and on up through the septum, splinting the ends together and taking the tension off the catgut. He has since taken the hint given by this case and adopted a similar procedure in six cases of complete tear of the perineum due to confinement. Two additional cases were operated upon by Dr. W. W. Russell and one by Dr. Ramsay. In each instance there was a surprising difference between the new and older methods, noted at once in the earliest stages of the convalescence, and the patient was immediately conscious of perfect control of her functions. The bowels should never be locked up. Great care must be taken not to leave any dead spaces in closing the remainder of the wound in the usual way, in order to avoid all risk of infecting the buried sutures. He only recommends this operation to those who possess considerable skill in doing plastic operations and in securing a snug, accurate adaptation of the parts.

In the discussion Drs. George H. Noble, of Atlanta, and J. Wesley Bovee, of Washington, D. C., added their testimony to the usefulness of the method described by Dr. Kelley, but certain features of the technique of which they modified.

Dr. Lewis S. McMurtry, of Louisville, Ky., read a paper on

THE TREATMENT OF CANCER OF THE UTERUS.

The Doctor referred to the statistics of vaginal hysterectomy as given by Pozzi in the third addition of his treatise on gynecology, an analysis of which does not strengthen or inspire confidence in the ultimate results of this procedure. He said that claims are being made for the permanent cure of uterine cancer by hysterectomy which could not be realized. Of all the cases of uterine cancer which apply for treatment, only a small proportion are within the scope of a clean extirpation by vaginal hysterectomy. The large number of cases in which the disease recurs at the site of operation within a few weeks demonstrates that the operation is in most cases simply a resection, leaving behind sufficient disease for continued activity. His personal experience with this operation has been discouraging. Vaginal hysterectomy for cancer has never been a favored operation with him. During the past year he has done the operation in five cases, which were selected as most favorable for permanent cure. In all the disease was discovered early, and, so far as microscopic evidence could show, it was limited to the uterus itself. The organ was normally mobile. Of these five cases treated by vaginal hysterectomy, two had recurrence, one in the bladder, the other in the vaginal fornix at the cicatrix, within five months after operation. These were selected cases in which the disease was early recognized; the patients were under 50 years of age and well nourished. The operation was done with a view of going far beyond the region of probable infiltration, and removing the appendages with the uterus. Based on his own previous experience, and that of other operators, it is doubtful if one of the three remaining cases will be living at the end of three years from the time of operation.

Dr. William L. Rodman, of Louisville, asked the members to give their experience relative to the frequency of cancer in the black and white races. He was rather surprised to hear of the infrequency of the disease in the negro woman. According to the last census statistics of Billings, cancer of the uterus is more common at all ages in the black than in the white race. This is also the experience of Matas, who has examined the records at the Charity Hospital in New Orleans. An examination of all deaths recorded by the Health Department of Louisville for the past thirty years corroborated the same view.

Dr. Earnest S. Lewis, of New Orleans, stated that while he had not observed a very marked difference in the relative immunity of negro women to cancer of the uterus, still if his experience is not at fault, he thinks, owing to their uncleanliness, their mode of living, and to the more frequent accidents to which they are subject during labor, they are particularly prone to cancer of the uterus. With regard to the results of all operations for cancer of the uterus, he endorsed everything Dr. McMurtry had said. He could only recall one case of vaginal hysterectomy for cancer that lived for eight years, after which the disease returned and the patient finally died. The disease is so liable to return that he considers any operation as palliative, and he believes that would be the result of the abdominal operation advocated by Dr. McMurtry, particularly in advanced cases, and if cases are met within the incipiency of the disease, in his opinion as much could be accomplished by the vaginal as by the abdominal operation.

Dr. Virgil Hardon, of Atlanta, said during his seven years' connection with the Grady Hospital, he had reason to believe that cancer of the uterus was more frequent in negro women than in white women. He has been led by his experience in the treatment of uterine cancer to the same conclusion as that reached by the essayist, except he had been led to go further. He has operated for cancer of the womb by vaginal extirpation, by the abdominal method, and by the combined method and he has never yet operated upon a case where recurrence did not take place sooner or later, and for this reason he has lost all confidence in operative measures as a means of effecting a permanent cure. However, he has no doubt on theoretical grounds, that if cases are seen sufficiently early, a permanent cure might be effected by surgical interference.

Dr. Howard A. Kelly, of Baltimore, remarked that he was astounded at the trend the discussion had taken, because he had seen dozens of cases of cancer of the uterus that had remained well for a number of years after having undergone surgical intervention, removing the uterus either by vagina or by the abdomen. He finds carcinoma of the uterus as frequently in negroes as in white women.

Dr. J. Wesley Bovee, of Washington, D. C., said that his experience in the radical treatment of cancer of the uterus has been more satisfactory than what he had been hearing to-day. He knows of a good many cases upon which he has operated that have gone on for three years or more without a recurrence of the disease. He has done three operations after the manner described by Werder in the American Journal of Obstetrics, last winter, and he is much pleased. In each case he adopted the abdominal rather than the vaginal route, believing a more radical operation could be performed by this method. He has great hope for the future treatment of cancer of the uterus by complete abdominal hysterectomy, undertaken early.

Dr. William L. Robinson, of Danville, said that when the parts have become infected beyond the uterus, no dissection, however extensive, will ever remove the cause. When the disease has extended beyond the uterus he believes it is beyond the power of any surgeon to thoroughly remove it. Therefore, unless an operation could be done very early, he could not advise the removal of the uterus.

Dr. W. E. B. Davis was profoundly impressed with the position taken by Dr. Lewis as to the ultimate outcome of cases of cancer of the uterus, believing his position is correct. There is no man in America who has had a larger experince in the treatment of cancer of the uterus than Dr. Lewis, and the profession were familiar with his great skill as a surgeon, hence his experience regarding uterine cancer was certainly valuable.

Dr. Murtry, in closing the discussion, said he could not endorse the view that Dr. Kelly takes with reference to the ultimate results of the operative treatment of cancer of the uterus. He knows that Kelly's work in this direction has been extensive, and that his reports are sincere and reliable, but there are a great many surgeons, working in the same line, who have done thorough, faithful, skillful work in the radical treatment of uterine cancer, who doubt the future confirmation of Dr. Kelly's views.

Dr. Joseph Tabor Johnson, of Washington, D. C., presented a paper on

### THE CONSERVATIVE TREATMENT OF THE DISEASED OVARY.

The difference between the radical and corservative treatment of the diseased ovary is somewhat difficult to define, inasmuch as the most radical treatment under some circumstances is really the most conservative, while in other cases, to conserve the best interests of some particular diseased ovary requires the most radical surgery.

In the early part of the present decade, quite a conservative wavelet swept over the country, and considerable harm was done to pelvic and abdominal surgery in the mild and gentle name of conservatism. Incomplete conservative operations were done, some of which had to be completed later on by radical operations. Some of the men who claimed to be the most conservative, and attracted the timid doctors and frightened patients, were actually removing more ovaries and tubes than many of their so-called radical friends. Much credit has been claimed for saving a part or the whole of one ovary and tube, when only a simple catarrhal salpingitis existed, by an operator posing before the profession and community as a conservative, when the surgeon designated a dangerous radical to be avoided would actually not have operated at all, and would probably have cured his patients by other means. In some instances genuine, successful conservatism was practiced with lasting beneficial results, but not always from the highest and purest motives. Again, it has been charged that actual radicalism has successfully masqueraded under the name and guise of conservatism to the injury of the trusting patient and the discredit of good surgery. So much has been learned by accumulating experience, as the domain of the gynecologist has undergone so much expansion, that real conservatism is gradually gaining ground over real radicalism to such an extent that he who presents ovaries and tubes or a fibroid uterus to a modern up-to-date medical society has to state very good reasons why he sacrificed these important organs in their entirety, to escape criticism and possibly censure. Sacrificial surgery is gradually giving way to more conservative and humane methods. He believes there is a maxim in general surgery in favor of saving every inch of the human body possible, and another that it requires a higher order of skill to save a mulilated or diseased member than it does to cut it off or to cut it out. With his present experience in abdominal surgery, he is free to confess that he can now save ovaries and tubes which he formerly thought it necessary to totally remove. The increasing skill of our abdominal surgeons and their accumulating experience in actual conservative work, go to show that we are approaching nearer to that true conservatism which is the offspring of increased skill and experience. In abdominal surgery it requires a higher order of skill and a greater experience to save an organ or part of an organ than it does to remove it. It is important to save a portion of one ovary, when the other has been removed on account of a tumor, an abscess, or for any other cause. The disagreeable symptoms accompanying the artificial and premature change of life are often stormy and protracted; in some rare instances threatening, if not resulting in actual insanity. These women are happily prevented from falling into such a condition by saving one or a portion of one ovary; menstruation is then not interrupted, and the sexual and other feelings of the patient undergo none of those sudden and peculiar revulsions which unfortunately sometimes follow the total removal of both ovaries and tubes. In conclusion, Dr. Johnson said that if growing experiences in the abdominal cavity and accumulation of evidence continue in favor of more conservative and less sacrificial operative work, he feels sure that the deep debt of gratitude now felt toward abdominal surgeons by suffering women will be tenfold increased and intensified.

Dr. Richard Douglas, of Nashville, Tenn., delivered the presidential address. He made a departure from the usual or stereotyped address and selected for his subject

### ACUTE GENERAL PERITONITIS.

Before considering his subject proper, he spoke of the growth of the association and the excellence of its scientific work, saying that the association owes its existence and high standing to the indefatigable efforts of Dr. W. E. B. Davis, the secretary.

A bacteriologic classification of peritonitis is beset with many difficulties, and while the speaker is free to admit that for all practical purposes peritonitis is of bacterial origin, yet there occurs a respectable percentage of cases in which the most rapid examination fails to disclose the presence of micro-organisms. The conclusions that may be drawn from an etiologic study of peritonitis may be thus summarized: Traumatic peritonitis, especially the post-operative variety, is essentially a grave condition, not only because there is immediate or primary inoculation of the peritoneum, but the conditions are all favorable for germ culture and dissemination. Peritonitis by continuity may become general and prove rapidly fatal, but this is not the rule except in puerperal cases. Contrary to the expressed opinion of more than one writer, there is nothing peculiar about the peritoneum, or the cecum, or appendix, or the true pelvis, which accounts for the more frequent localization of inflammation in these regions than in other areas of the abdomen. The method of invasion, the activity of the process and resistance of patient alone determine the local or general type of peritonitis. Visceral perforation, whether traumatic or pathologic, is an ideal condition for germ culture and the elaboration of toxins. Their rapid absorption and general diffusion throughout the peritoneum sufficiently explain the grave state into which the patient is precipitated. It may be asserted that the clinical course and pathologic expression depend largely upon the nature of the exciting cause, the character of the pre-existing disease or injury, and the mode of invasion.

Without attempting to formulate any definite pathologic classifications of general peritonitis, and to adapt each to its special course, he prefers to direct his efforts to simplifying and dispelling the confusion that exists. He accepts, with slight reservation, the now almost universally conceded idea that acute general peritonitis is and must be septic, that is, of bacterial origin. But he maintains that surgeons do not understand each other, nor have they all a clear conception of what is meant by septic peritonitis. It has been a race for life between the practical surgeon and bacteriologist as to who should claim the honor of naming the pathologic phenomena in this great serous bursa. He maintains that the surgeon is alone competent to define, classify and prognosticate the protean types of this disease. That men are honest, painstaking and accurate, goes without saying, yet how can surgeons reconcile the report of McCosh's last series of eight cases of general septic peritonitis and six operative recoveries with the experience of Senn "of many cases of diffuse septic peritonitis without a single successful result." The answer certainly is not in the superior skill or special technique of the operator, but it is to be found in an analytic study of McCosh's cases, six of them were purulent peritonitis and two serous peritonitis, not the class of cases referred to by Senn at all. Tietze defines diffuse septic peritonitis to be "that form of peritonitis in which there is little or no exudate, severe symptoms of intoxication and terminating rapidly fatally." Some four years ago, Dr. Douglas reported eight cases of general peritonitis subjected to operation with four recoveries and four deaths. He dealt with them all as cases of general septic-peritonitis from a misconception of the term. Two of them only properly belonged to this class, and they terminated fatally. The others were cases of general purulent peritonitis with two deaths and four recoveries. He is very well pleased with his percentage of recoveries, but disgusted with his classification. He did not discriminate between diffuse septic peritonitis, the patient dying in twelve hours with profound toxemia and dry peritoneum after perforation of the appendix, and one of perforation with enormous purulent effusion, but mild symptoms of sepsis. This error of his is the common one with the profession and it is the outcome of an attempt to classify peritonitis by the character of exudate.

---

PUERPERAL ECLAMPSIA.—Baron and Castaigne have carried out (*Arch de Med. Exper.*, September 1898) an important series of experiments with a view to proving the fœtal origin of puerperal eclampsia. Within recent years pathologists have come to the conclusion that puerperal eclampsia is not due to maternal, renal or hepatic diseases, at any rate wholly, but that absorption of toxic products from either the fœtus or its annexæ, as to whether the fœtus or the amnion is most to blame, forms the subject of the writers' investigations. They find that certain substances injected directly into the fœtus or the amnion are rapidly absorbed by the maternal organism, provided the fœtus is living, but much more rapidly from the fœtus than from the amnion. From this it would seem that the fœtus secretes certain toxic substances into the blood and amniotic fluid. What these toxins are is to form the subject of a subsequent investigation. Secondly, if the fœtus be dead, substances injected into either amnion or fœtus do not seem to pass into the maternal circulation. This would seem to throw considerable light upon the various phenomena of eclampsia, and especially as showing that the death of the fœtus is followed by cessation of the convulsive seizure.

